# Acriflavine, an Acridine Derivative for Biomedical Application: Current State of the Art

**DOI:** 10.1021/acs.jmedchem.2c00573

**Published:** 2022-08-26

**Authors:** Kinga Piorecka, Jan Kurjata, Wlodzimierz A. Stanczyk

**Affiliations:** Centre of Molecular and Macromolecular Studies, Polish Academy of Sciences,Sienkiewicza 112, 90-363 Lodz, Poland

## Abstract

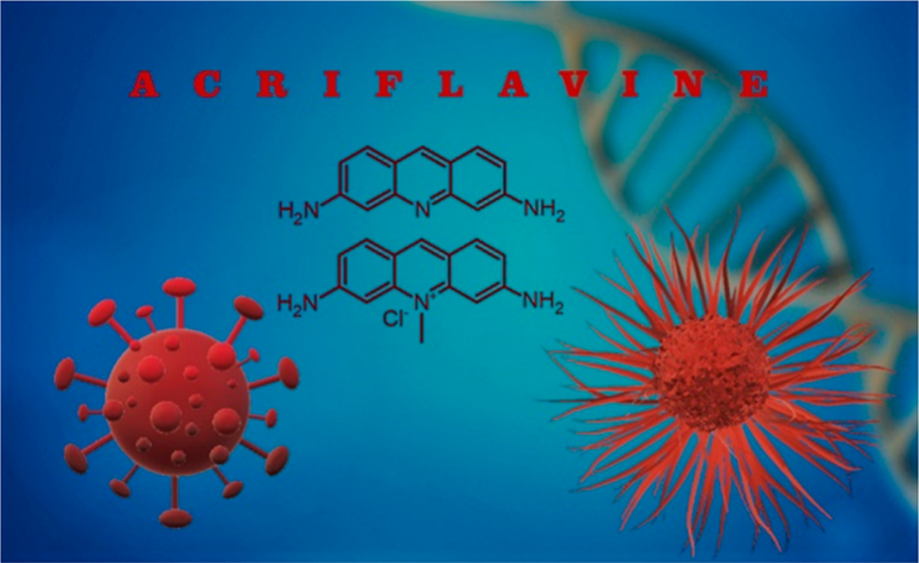

Acriflavine (ACF) has been known for years as an antibacterial drug. The identification of key oncogenic mechanisms has brought, in recent years, a significant increase in studies on ACF as a multipurpose drug that would improve the prognosis for cancer patients. ACF interferes with the expression of the hypoxia inducible factor, thus acting on metastatic niches of tumors and significantly enhancing the effects of other anticancer therapies. It has been recognized as the most potent HIF-1 inhibitor out of the 336 drugs approved by the FDA. This work presents up-to-date knowledge about the mechanisms of action of ACF and its related prodrug systems in the context of anticancer and SARS-CoV-2 inhibitory properties. It explains the multitask nature of this drug and suggests mechanisms of ACF’s action on the coronavirus. Other recent reports on ACF-based systems as potential antibacterial and antiviral drugs are also described.

## Introduction

**Figure 1:**
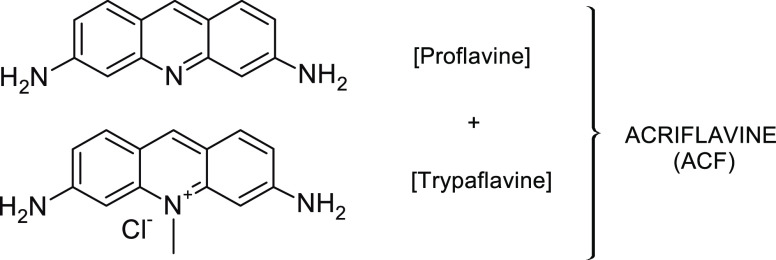
Chemical structure of acriflavine (ACF).

Acriflavine (ACF) is an acridine dye, first synthesized in 1912 by German scientist Paul Ehrlich and recognized as one of the first used antibacterial drugs, which was later replaced upon the discovery of penicillin. It was used extensively during World War I as an antiseptic and for treatment of coma. In addition, it has been also approved by the U.S. Food and Drug Administration (FDA) as a safe drug for the topical treatment of wounds.^1^ ACF is a mixture of 3,6-diamino-10-methylacridine chloride (trypaflavine) and 3,6-diaminoacridine (proflavine) (Figure 1).^2^ Its biological activity is attributed to the fact that it effectively intercalates with deoxyribonucleic acid (DNA).^3−6^ As a result, it has the ability to interfere with many cellular functions. ACF is a multidirectional drug, as it acts as an inhibitor of protein kinases, topoisomerases I and II, and hypoxia-induced factor 1α (HIF-1α) and reduces the expression of oncogenic STAT5 signaling.^1^ ACF is a potent epithelial-to-mesenchymal transition (EMT) inhibitor that lowers metabolic pathways, especially the mitochondrial oxidative phosphorylation system (OXPHOS) and MYC/cell proliferation,^7,8^ blocks eukaryotic initiation factor 2α (eIF2α) phosphorylation, and reduces activating transcription factor 4 (ATF4) translation by inhibiting the PERK/eIF2α/ATF4 UPR pathway^9^ and AKT and RSK2 phosphorylation.^10^ ACF also leads to upregulation of genes, especially long non-coding ribonucleic acids (lncRNAs).^11^

Recently its antimalarial,^12^ antibacterial,^13^ antiviral (HIV),^14^ antituberculosis,^15^ fungicidal,^16^ and anticancer activities have been recognized.^17^ Currently, ACF has been suggested as a potential drug for SARS-CoV-2, showing activity against the PL^pro^ enzymes involved in the reproduction of the coronavirus.^18^

Its anticancer effects deserve particular attention. It was first described over 60 years ago,^19^ but the breakthrough came only in 2009 with the research published by the research group of Gregg L. Semenza.^17^ Several mechanisms of ACF antitumor activity have been proposed, related to inhibition of topoisomerases I and II and HIF-1α. HIF-1α factor determines the aggressiveness of the tumor; therefore, its destruction may have a significant antitumor effect. ACF is also involved in inactivating this factor, which is an important action in therapy against the SARS-CoV-2 coronavirus. ACF sensitizes drug-resistant cancer cells; therefore, its effectiveness has been proven in combination therapy with other drugs to which the body has already developed resistance. It was indicated that ACF constitutes the most potent HIF-1 inhibitor out of the 336 FDA-approved drugs.^17^ Currently, ACF has been shown to be effective against a broad spectrum of cancers (osteosarcoma, breast, brain, lung, liver, colon, ovarian, and pancreatic cancers, and leukemia). The pharmaceutically significant factor is that ACF shows no side effects even when used extensively for several months.^20^

In addition to its anticarcinogenic role, ACF is being applied in other fields, e.g., for the development of a DNA sensor,^21^ a semiconductor biosensor for the detection of Sudan I–IV azo dyes,^22^ and a biosensor for detection of staphylococcal enterotoxin B (SEB),^23^ for treatment of seawater,^24^ as well as in optoelectronics and solar cells,^25,26^ as a contrast agent for imaging the upper- and lower-GI mucosa,^27^ and for determining drug concentrations (e.g., ketoprofen, diclofenac sodium, olsalazine).^28,29^

This Perspective concentrates on the current knowledge on the anticancer properties of ACF as well as its effectiveness against SARS-CoV-2.

## 1 Formation of Cancer

Cancer is a multistage process involving the uncontrolled growth of cells and inactivation of apoptotic mechanisms as a result of the integration of a tumor microenvironment composed of immune, stromal, and vascular cells. This process begins in a single mutant cell and is usually associated with the activation of oncogenes and the inactivation of suppressor genes.^30−32^ Normal tissue homeostasis is disrupted as a result of factors such as cytokines, and tumor growth factors are secreted. Tumor progression is related to the tumor stroma, an important component of which are innate immune cells (macrophages, dendritic cells, neutrophils, NK cells, innate lymphoid cells, myeloid suppressor cells) and acquired immunity cells (T and B lymphocytes). Cytokines in the tumor microenvironment influence immune functions, suppressing immune responses.^33^

### 1.1 Angiogenesis

Neoplastic cell proliferation may become limited as it requires the supply of oxygen and nutrients and removal of waste products. Therefore, angiogenic processes are activated in order to create blood vessels in the tumor microenvironment from the host’s capillaries. The process of angiogenesis begins after neovascularization, i.e., destabilization of the membrane that protects the endothelial cells. Then, these cells are activated by angiogenic factors, thanks to which they gain migratory, proliferative, and stabilizing abilities to create new immature blood vessels.^34,35^ Angiogenesis is regulated by numerous signaling pathways (including VEGF, HIF, PDGF, SDF-1, CXCR4, and MMP9) and by the balance between activators and inhibitors (Table 1).^36^

**Table 1:** Endogenous Regulators of Angiogenesis

activators	inhibitors
VEGF – vascular endothelial growth factor family	IL-10 – interleukin-10
aFGF, bFGF – acidic and basic fibroblast growth factors	IL-12 – interleukin-12
TGF-β – transforming growth factor β	TIMP – tissue inhibitor metalloprotease
TNF-α – tumor necrosis factor α	PAI-1 – prasminogen activator-inhibitor-1
PDGF – plated-delivered endothelial growth factor	zinc
HGF – hepatocyte growth factor	Ang2 – angiopoietin-2
placental growth factor	angiotensin
GM-CSF – granulocyte-macrophage colony-stimulating factor	AT2 – angiotensin-2
angiogenin	CAV-1, CAV-2 – caveolin- and -2
IL-1 – interleukin-1	endostatin
IL-6 – interleukin-6	INF-α – interferon-α
IL-8 – interleukin-8	platerat factor 4
cathepsin	
MMP9 – matrix metallopeptidase 9	
copper	
CD51/CD61 antibodies – alpha 5 beta 3 integrin angiopoitin-1	
AT1 – angiotensin-1	
endothelin	
erythropoietin	
HIF-1α – hypoxia-inducing factor	
NO – nitric oxide	
plated-activating factor	
PGE – prostaglandin E	

### 1.2 Hypoxia

Oncological treatment of a tumor involves not only impeding the development of the blood vessel network but also destroying cancer cells that survive malnutrition, hypoxia (lack of oxygen),^37^ and immune cell attacks. Cancer cells are equipped with appropriate mechanisms to avoid antitumor immune responses and can develop mechanisms of adaptation to hypoxia, as a result of which the hypoxia-inducible factor (HIF-1) is activated. HIF-1 is a transcription factor composed of α (HIF-1α, HIF-2α, and HIF-3α) and β subunits.^38^ Under hypoxic conditions, HIF-1α is stable and interacts with HIF-1β, resulting in the formation of a heterodimer that induces the transcription of many genes, regulates the expression of factors involved in tumor metabolism and vascularization, and activates the expression of the factor promoting angiogenesis (VEGF), as well as glucose transporters (e.g., GLUT-1) and glycolytic enzymes (e.g., hexokinase) that are required for high levels of glucose absorption and metabolism.^39,17^ In addition, HIF-1 is involved in the maintenance of cancer stem cells (CSCs) that are self-renewing, chemically resistant, and involved in metastasis and promoting EMT.^40^

HIF-1 plays the key role in activating more than 100 genes that regulate glucose metabolism (Warburg effect), cell proliferation, migration, and angiogenesis. It promotes metastasis through the transcriptional activation of oncogenic growth factors (TGF-β, EGF). Activation of the major hypoxic factors (HIF-1) supports the creation of a cancer-promoting microenvironment. Hypoxia mainly affects solid tumors; however, pancreatic cancer differs from most solid tumors in its high stromal content, and therefore it is characterized by a particular hypoxia and is able to survive in a changed microenvironment thanks to the mechanisms of interaction between pancreatic cancer cells and stromal cells and the activation of many signaling pathways, such as AKT, STAT3, and ERK.^41^

Severe hypoxia, oxidative stress, and endoplasmic reticulum stress engage additional signaling pathways such as unfolded protein response (UPR) that leads to inhibition of eIF2α through phosphorylation and activation of EMT-associated ATF4 and drug resistance.^9,42^

Hypoxia also leads to the induction of reactive oxygen species (ROS) which is involved in the activation of poly[ADP-ribose]polymerase 1 (PARP-1), which stabilizes and activates HIF-1α.^43^ HIF-1α up-regulates PDK1 and increases glucose uptake by GLUT1 transporters.^44^ HIF-1α can specifically induce increased expression of lysyl oxidase (LOX) and glycolytic enzymes. HIF-2α, on the other hand, is involved, *inter alia*, in the TGF-α, Oct-3/4, and Sox-2 pathways.^45^ HIF-1α can also be activated under non-hypoxic conditions. It is possible through activation of the PI3K/AKT/mTOR pathway in which eIF-2α participates.^10,46^

### 1.3 Epithelial-to-Mesenchymal Transition (EMT)

**Figure 2:**
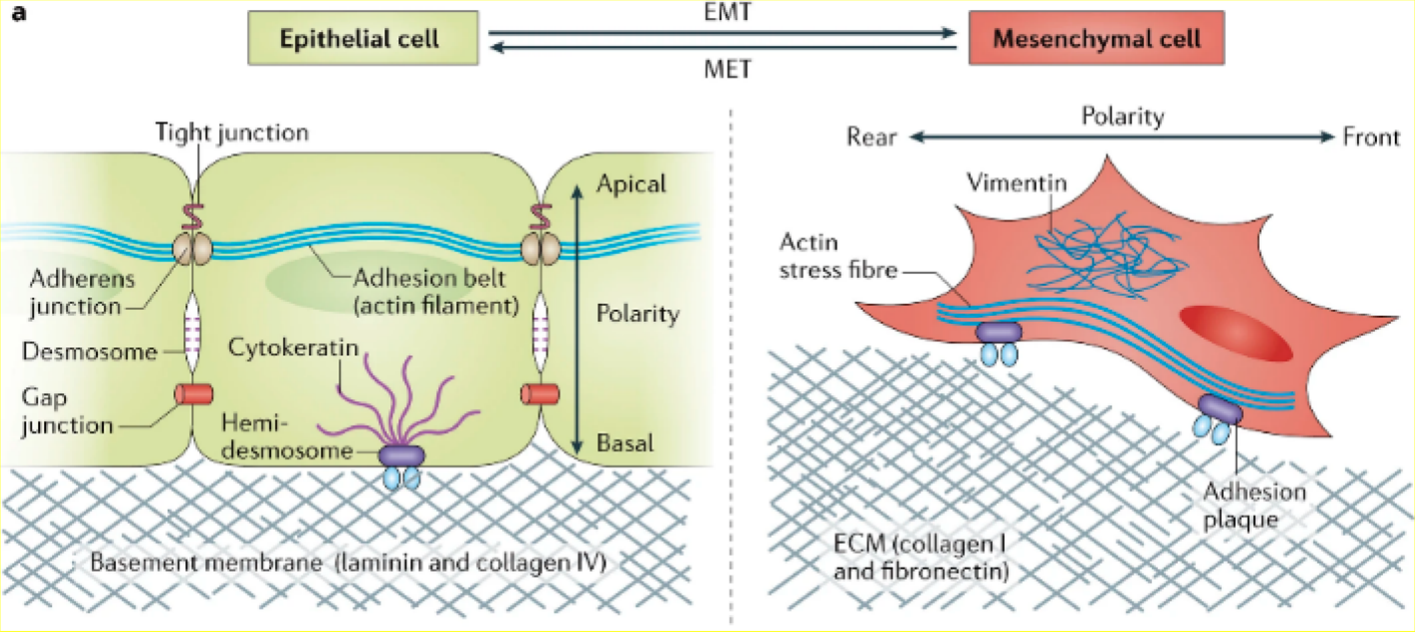
Morphological and physiological changes associated with the epithelial-to-mesenchymal transition (EMT). Reprinted with permission from ref (60). Copyright 2017 Springer Nature.

Formation of new tumor blood vessels and the mutual regulation of neoplastic and stromal cells enable the promotion of metastasis. The metastasis process is related to the activation of EMT, which gives cancer cells the ability to migrate and further invade.^47,48^ In this process, changes in cell morphology and physiology occur: cells lose their epithelial features, polarization, and E-cadherin-dependent intercellular junctions (dependent on the expression of vascular endothelial growth factor (VEGF) and epidermal growth factor receptor (EGFR)).^49,50^ As a consequence, cells acquire a mesenchymal phenotype. As a result, these cells acquire migration properties that allows them to move to other places in the body. These cells then go through the opposite process, called the mesenchymal-to-epithelial transition (MET), settle down, and form metastases (Figure 2).^51−53^ Multiple signals from the tumor microenvironment can initiate EMT, including TGF-β, HIF-1α, epidermal growth factor (EGF), WNT, and Notch.^52^ The course of EMT is also influenced by other cytokines, including hepatocyte growth factor (HGF) and fibroblast growth factor (FGF).^52^ Studies have shown that EMT is associated primarily with the activation of the transforming growth factor beta (TGF-β)/Smad pathway, causing upregulation of EMT-promoting transcription factors (Snail, Twist, Slug, and ZEB); epithelial gene expression is suppressed in favor of activation of mesenchymal gene expression.^54,55^ Such activities favor the formation of metastases related to the mechanisms of cytoskeleton reorganization, basement membrane degradation (through activation of matrix metalloproteinases (MMPs)), and avoiding apoptosis.^56,57^ Recent studies have shown that not all cancer cells undergo the full EMT process or gradually acquire mesenchymal features. This applies to such cancers as breast, kidney, colon, lung, and pancreatic, among others.^58,59^ There are also cancer stem cells (CSCs) in the EMT cell population that exhibit cellular characteristics similar to those of EMT cells. It has been reported that there is an association between EMT and CSCs promoting drug resistance and tumor malignancy.^47,60,61^

## 2 Physical and Biological Properties of Acriflavine

ACF is a mixture of trypaflavine (C_14_H_14_ClN_3_, molar mass = 259.74 g·mol^–1^) and proflavine (C_13_H_11_N_3_, molar mass = 209.25 g·mol^–1^), which mutually stabilize each other.^1,12^ The water solubility of ACF makes it potentially injectable. ACF is a planar molecule containing three aromatic rings with a polycyclic arrangement.^13^ The planar layout and the positive charge allow ACF to intercalate between nucleotide base pairs in the DNA helix.^62−66^ Proflavine, as a component of ACF, has a recognized role in the intercalation of DNA, and its activity is based on the mechanism of release of ROS, which was described by N. Imrana’s team. Proflavine changes the structure of the DNA strand and binds to topoisomerases I and II, loosening or splitting the double-stranded DNA and intercalating between adjacent layers of nucleotide pairs.^11,67^ This leads to a series of mutations in the genetic material or apoptosis. Proflavine has been found to bind better to alternating purine–pyrimidine DNA sequences than ACF.^63,64^ Other studies highlight the toxicity of ACF after exposure to light at 448 nm, also related to the induction of DNA damage.^68^ In addition, adding ACF to the treatment of infections of the urinary tract with methanamine and methylene blue resulted in an increase in the number of side effects.^69^ There are also reports of the effectiveness of ACF (e.g., against HIV1) with little to no toxicity.^14,70^

ACF is an effective inhibitor of HIF-1α aimed primarily at the treatment of solid tumors.^39^ ACF interferes with HIF-1α (or HIF-2α) dimerization with HIF-1β, inhibiting the transcriptional activity of HIF-1.^17,39^ ACF also sensitizes drug-resistant cancer cells by inhibiting EMT. It has has also been shown to be effective in, e.g., treating chronic myeloid leukemia (CML) and acute myeloid leukemia (AML).^71^ It exhibits anti-neoplastic activity against a broad spectrum of cancers, including colorectal,^38,72^ periapharyngeal bile duct,^73^ breast,^74^ pancreatic,^7^ liver,^75^ cervical,^76^ and brain cancers^39^ and melanoma.^10^

The use of many anticancer drugs can increase HIF-1α levels as a result of increased levels of ROS in cancer cells. HIF-1 inhibition by ACF can increase the effectiveness of these drugs by preventing chemoresistance. In addition, ACF may facilitate the penetration of chemotherapeutic agents because it binds to the cell surface membrane and leads to the inhibition of protein kinase C.^72^

ACF is more effective than other inhibitors of factors involved in tumor cell proliferation (e.g., VEGF, GLUT-1, PD-L1) because a greater antitumor effect can be achieved by direct inhibition of HIF-1 rather than by inhibiting, e.g., VEGF.^17^ When targeting VEGF, HIF-1α will further dimerize with HIF-1β to form HIF-1 and re-initiate downstream gene transcription.

**Figure 3:**
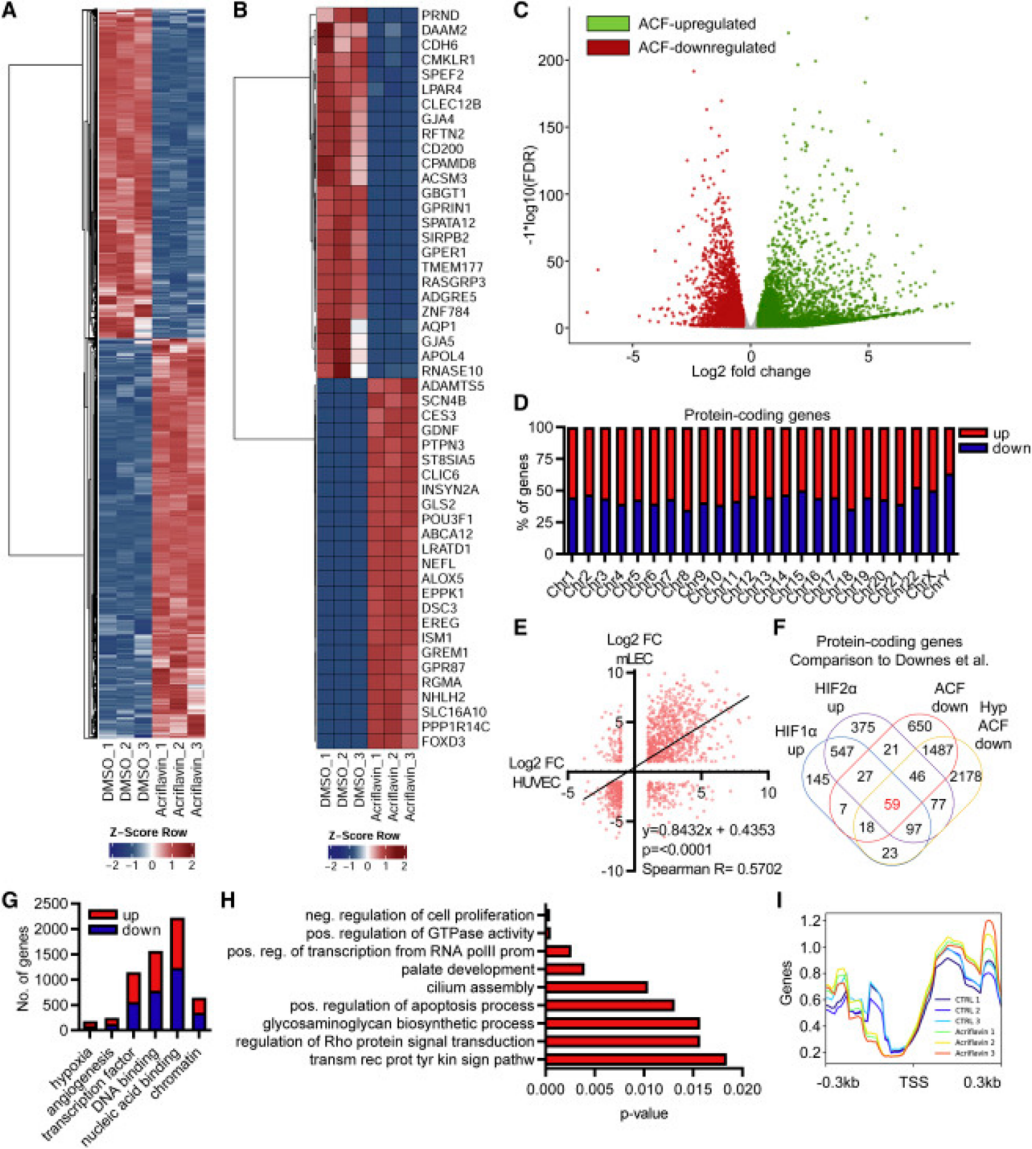
Acriflavine (ACF) strongly changes gene expression. (A, B) Heatmaps of RNA-seq after treatment of HUVECs with ACF. (C) Volcano plot of RNA-seq after treatment of HUVECs with ACF. (D) Chromosomal distribution and percentage of protein-coding genes up- or downregulated with ACF. (E) Correlation analysis of protein-coding genes up- or downregulated with ACF of HUVECs and murine lung endothelial cells. (F) Venn diagram. (G) Number of protein-coding genes up- or downregulated with ACF. (H) GO enrichment analysis with KOBAS2.0. (I) deeptools2: overlaying the RNA-seq reads with the transcription start sites of all genes. Reprinted with permission from ref (11). Copyright 2022 The Authors. Published Open Access by Elsevier under a Creative Commons CC BY license.

Recent studies (RNA sequencing, Figure 3) show that treating endothelial cells with ACF leads to a strong change in the expression of hundreds of genes, regardless of normoxia or hypoxia, and that it elicits a unique expression response of long non-coding RNAs (lncRNAs). These studies also suggest that the mechanism of action of ACF on endothelial cells may not be related to HIF inhibition, as only about 10% of ACF-responsive genes have been shown to be HIF-dependent. ACF promotes topoisomerase inhibition independently of HIF-1. This effect is different from the effect on cancer cells that are more sensitive to ACF. But ACF has been suggested as a link between lncRNA expression and cancer therapy.^11^

Other RNA sequencing data are also available that provide information on EMT regulation and metabolic pathways following ACF use.^7,8^ This study shows that ACF downregulates metabolic pathaways, especially OXPHOS and MYC/cell proliferation pathways in pancreatic cancer xenografts.

## 3 Anticancer Properties of Acriflavine

ACF was found to be more effective against HCC liver cancer than the currently used sorafenib (see Table 3, below).^75,77^*In vitro* studies have shown that the IC_50_ of ACF (1 μM) is almost 10 times lower than the IC_50_ of sorafenib (13.4 μM). In an animal model, ACF treatment has been shown to reduce tumor size in nude mice.^75^

ACF enhances the antitumor activity of sunitinib in a breast cancer model^78^ and of 5-fluorouracil used in the treatment of colorectal cancer much better than irinotecan.^79^ It acts on HIF-1 by reducing the expression of LOX and LOXL proteins (responsible for metastases), destroying the metastatic niches of breast cancer.^80^ It was also proven that the synergistic effect of ACF and ABT-263 drugs strongly exerted triple negative breast cancer (TNBC) apoptosis. The action of these drugs compensated for each other by inhibition of BCL-2, BCL-XL, and BCL-1 due to the action of ABT-263 and by inhibition of MCL-1 independently of the HIF-1 pathway with ACF.^81^

It was shown that ACF loaded into poly(lactic-*co*-glycolic acid) (PLGA) microparticles resulted in an *in vitro* release of the drug for up to 60 days, which may be of importance in the treatment of choroidal neovascularization (NV).^82^ The cause of this disease is, among others, hypoxia resulting from the action of HIF-1 and HIF-2. Unlike PLGA-ACF microparticles, free ACF is potent but short-lived because, as a small molecule, it is quickly cleared from the eye. *In vivo* studies showed that intravitreal injection of the PLGA-ACF MPs complex with ACF in mice inhibited choroidal NV for at least 9 weeks. Moreover, supravascular injection of these microparticles in rats inhibited choroidal NV for at least 18 weeks.

**Figure 4:**
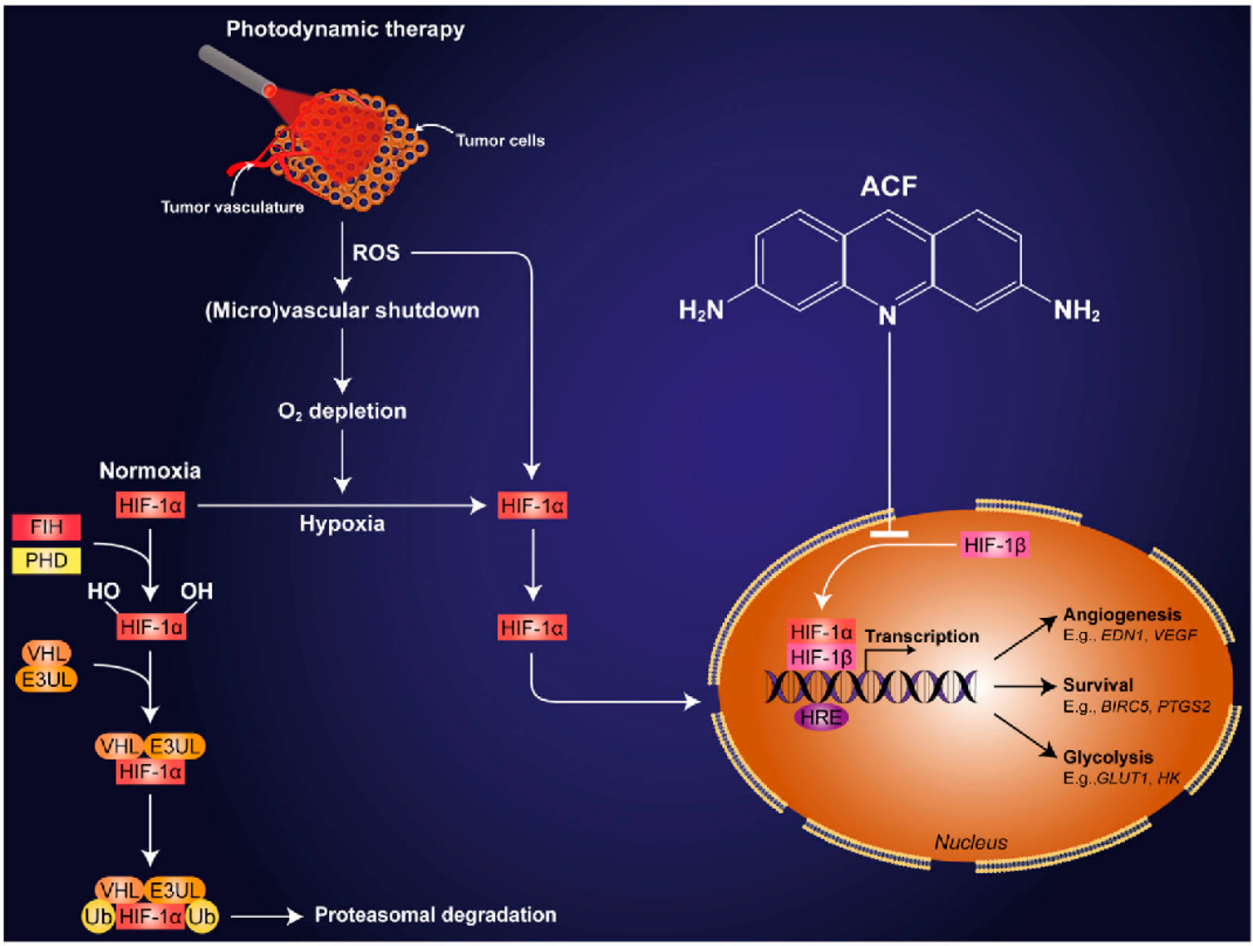
Diagram of ACF action in photodynamic therapy. Reprinted with permission from ref (86). Copyright 2016 Springer Nature.

The necessity of encapsulating ACF was also highlighted by comparison of the action of free ACF with that of ACF loaded in lipid nanocapsules (LNCs).^83^ The higher antitumor efficacy of ACF-loaded nanoparticles in an orthotopic mouse model of breast cancer (4T1 cells) was confirmed as a result of HIF-1 inhibition. This led to a reduction in the number of drug administrations from 12 to 2. It was also shown that paclitaxel (PTX) was more effective against cancer-associated fibroblasts (CAFs) when encapsulated in LNCs.^84^ The recent studies by Morteza Eskandani’s research group also suggested the need to incorporate ACF into solid lipid nanoparticles (SLNs).^85^ ACF-SLN cytotoxicity studies against A549 human epithelial carcinoma cells showed that ACF-SLN was more effective than free ACF. It turned out that the use of ACF in photodynamic therapy (PDT) with liposomal zinc phthalocyanine enhanced the therapeutic effect (Figure 4).^86^ PDT is a minimally invasive method of treating various solid neoplasms, leading to accumulation of a photosensitive drug (photosensitizer) in the tumor that, on irradiation, is activated, generating ROS. It causes a state of stress hyperoxidation and, as a consequence, leads to the death of neoplastic cells. PDT leads to tumor hypoxia, but it may be ineffective for tumors that have developed a hypoxic survival system associated with the activation of HIF-1 and the promotion of the transcription of genes encoding P-glycoprotein.^87^ Consequently, the tumor is resistant to PDT. This is often the case of bile duct cancer of the nasopharynx and epidermal cancer. Therefore, the introduction of ACF as a HIF-1 inhibitory drug leads to better results of PDT and death of human epidermal carcinoma (A431) cells^86^ and perihilar cholangiocarcinoma (SK-ChA-1).^73^

**Figure 5:**
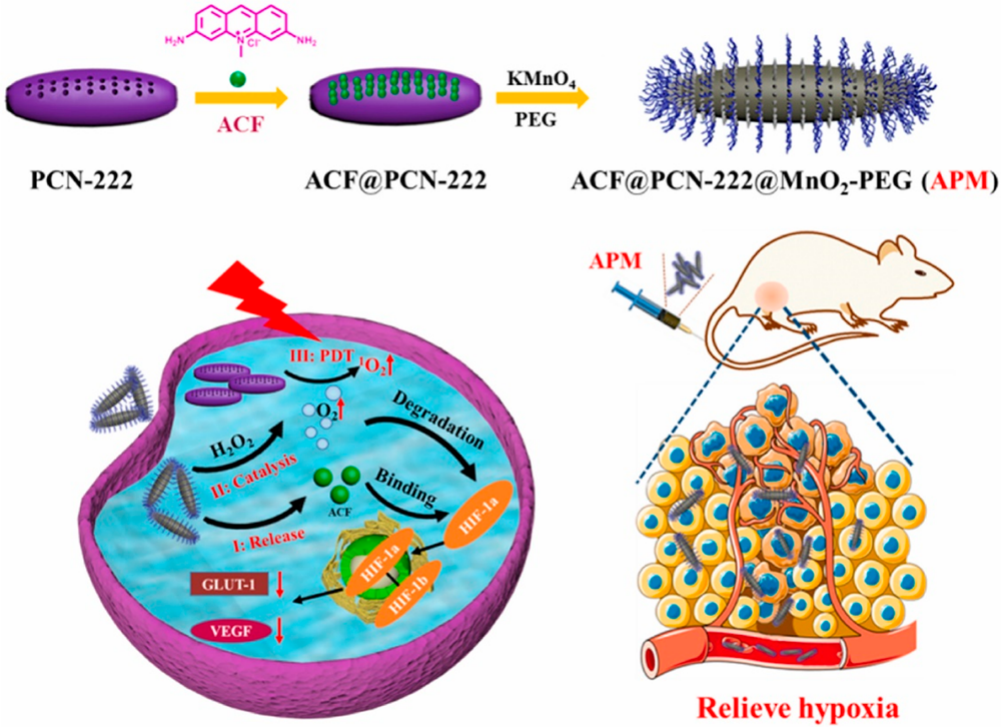
Scheme of the synthesis of the ACF@PCN-222@MnO_2_-PEG nanoplatform and its anticancer activity in photodynamic therapy. Reprinted with permission from ref (76). Copyright 2021 Elsevier.

The created multitask nanoplatform based on ACF, porphyrins, and manganese dioxide (ACF@PCN-222@MnO_2_-PEG) effectively reduced the expression of HIF-1α, and then GLUT-1 and VEGF, which gave anticancer effects both *in vitro* and *in vivo*. The action of this system is related to the joint operation of both the PCN-222 nanoparticles, reducing the self-quenching of porphyrins and increasing the ability to produce singlet oxygen, and ACF. The latter can be released in a controlled manner, depending on the H_2_O_2_ overexpression in the tumor, as the MnO_2_ layer on the surface of the carrier decomposes into Mn^2+^ and O_2_, releasing the drug.^76^ Additionally, the oxygen released during the decomposition of MnO_2_ may promote the effect of PDT against hypoxia (Figure 5).

Another example presenting evidence for ACF activity in PDT was provided by the release system involving zinc(II) phthalocyanine (ZnPc), ACF, and Fe^3+^. Fe^3+^ catalyzes the conversion of H_2_O_2_ → O_2_, promoting the synergistic activity of ZnPc and ACF in *in vitro* tests against HT29 cells and *in vivo*.^88^

**Figure 6:**
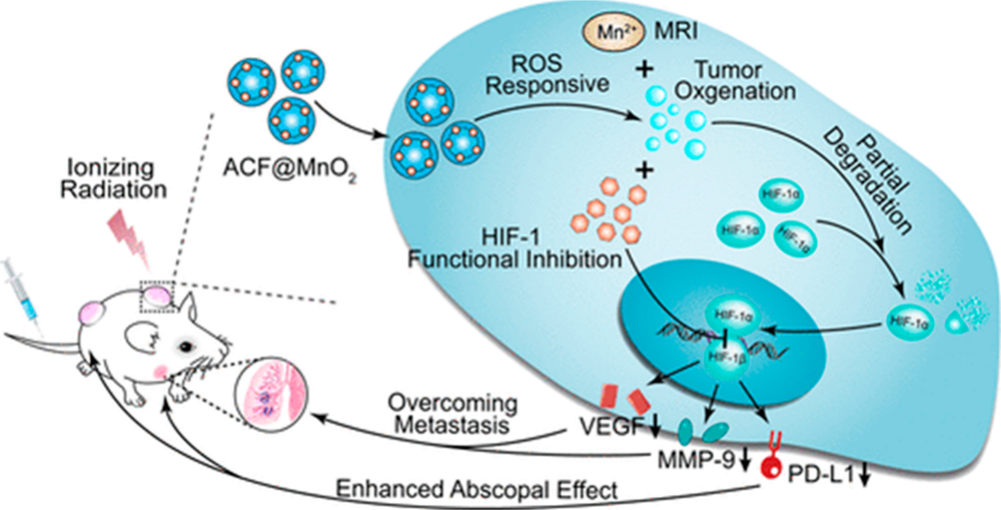
Use of the ACF@MnO_2_ nanoplatform to enhance radiotherapy. Reprinted with permission from ref (92). Copyright 2018 ACS.

It is also possible to enhance the effect of radiotherapy in cancer treatment by using ACF. In radiotherapy one encounters problems due to resistance induced by hypoxia.^89^ To counteract this, several methods have been developed, including the method of oxygen supply. Unfortunately, this method is not fully effective, as it does not induce complete degradation of HIF-1α due to the rapid consumption of oxygen by proliferating cancer cells.^90,91^ Even small amounts of HIF-1α will dimerize with HIF-1β to form HIF-1. Therefore, the use of ACF may be of key importance to enhance the effect of radiotherapy. A nanoplatform was synthesized consisting of MnO_2_ and ACF, which enhanced the effect of radiotherapy and significantly reduced metastatic lesions in lung and liver tissues (Figure 6).^92^

The importance of using ACF in radiotherapy was further confirmed by studies exploiting the new type of yolk–shell Cu_2–*x*_Se@PtSe (CSP) nanosensitizer functionalized with ACF.^74^ Electrostatic drug–vehicle interactions were shown to be involved in tumor cell cycle arrest, making cancer cells more susceptible to X-rays.

ACF can be helpful not only in radiotherapy but also in chemotherapy. As in PDT, cytostatics therapy increases the level of ROS in tumors, HIF-1α stabilizes, and the level of proteins associated with resistance (glycoproteins, GLUT-1, MMP-9) increases, which in turn leads to drug resistance. An example of therapeutic resistance is evident in the use of DOXIL (FDA approved in 1995) for treatment of cancer, which was found to be an improvement over the use of free doxorubicin (DOX), which caused cardiotoxicity.^93^ The formation of ROS and consequently the development of drug resistance after the use of DOXIL are associated with the formation of semiquinone in the DOX ring system.^94^ To counteract this, the use of ACF in liposomal DOX chemotherapy has been described, and formation of a DOX-ACF@Lipo complex turned out to be effective in the treatment of colorectal cancer.^95^ ACF has been encapsulated in the hydrophilic core of the lipid bilayer together with DOX.

Microporous silica-coated cisplatin nanoparticles with absorbed ACF were found to inhibit HIF-1, which led to increased antitumor efficacy against A549 lung cancer cells both *in vitro* and *in vivo*.^96,97^

**Figure 7:**
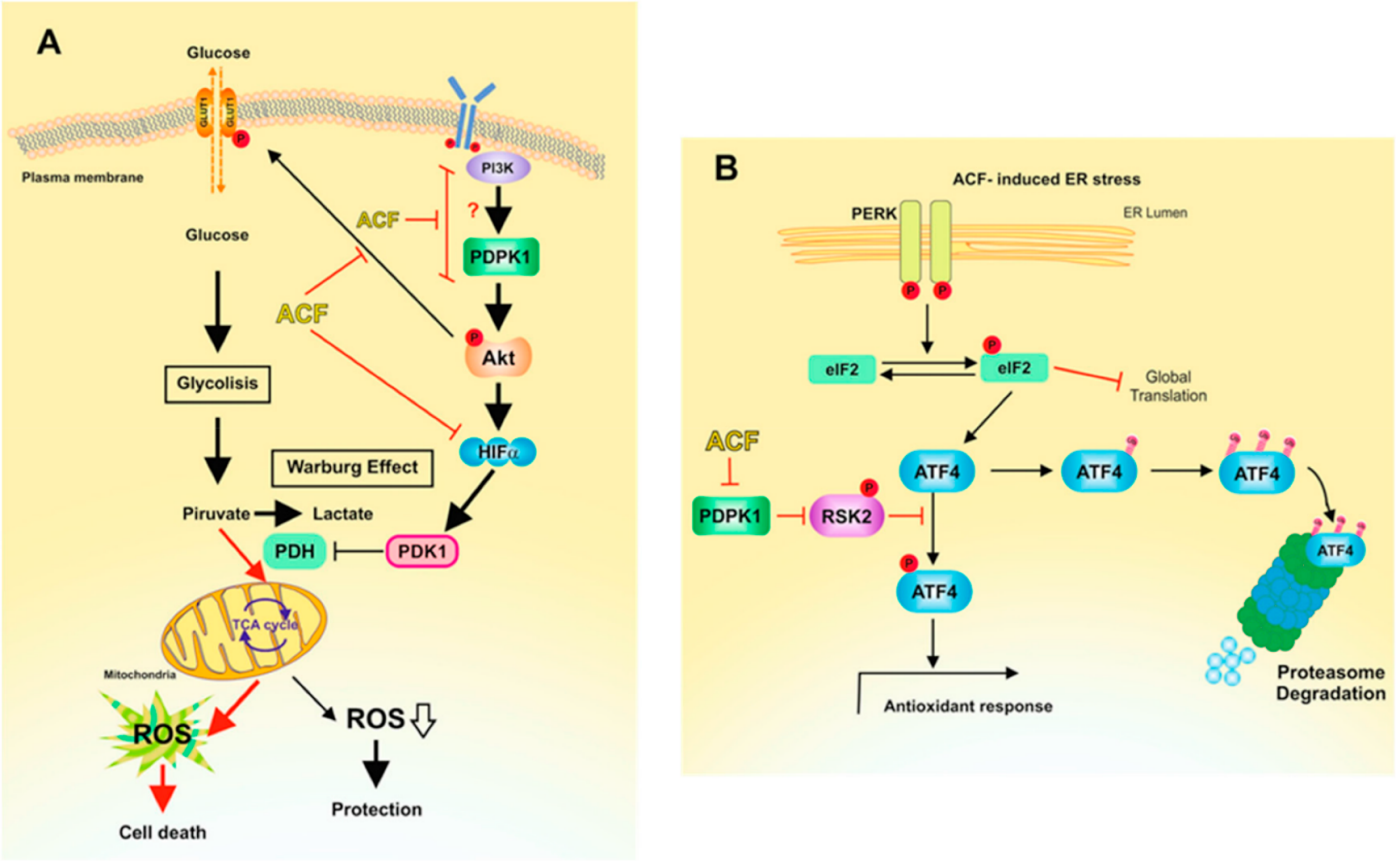
Mechanism of ACF’s action on melanoma under normoxic conditions. (A) ACF inhibits AKT phosphorylation. (B) ACF inhibits the phosphorylation of ATF4, mediated by RSK2. Reprinted with permission from ref (10). Published 2020 Open Access by MDPI.

Apart from hypoxia, also in normoxia, ACF exhibited high antitumor activity. Under normoxic conditions, HIF-1α levels are low and proteasomal degradation of HIF-1α occurs, but there are other stimuli capable of expressing HIF-1 in tumors under these conditions. These are, for example, cytokines or TLR proteins that induce tumor progression. Therefore, it is important to use ACF in order to inhibit not only TLR3 signaling but also HIF-1, leading to increased effectiveness in breast cancer treatment.^98^ Melanoma can also activate hypoxia response pathways even under normoxic conditions, indicating the participation of HIF-1α enabling survival under oxidative stress.^99^ The influence of ACF on the metabolism and progression of melanoma under normoxic conditions was described (Figure 7).^10^ It was proven that inhibition of HIF-1α with ACF in melanoma can be an effective cure against this tumor, regardless of the tumor’s hypoxic state. The proliferation of melanoma cells under conditions of reduced glucose concentration causes activation of a rescue path, i.e., an increase in the expression of the transcription factor ATF4, which is involved in cancer progression and resistance to therapy.

ACF inhibits the phosphorylation of RSK2 in the PDPK1/RSK2 pathway, which destabilizes ATF4. As a result, it weakens the effect of ATF4, leading to degradation of proteasomes (Figure 7B). Glucose restriction also activates the AKT pathway in melanoma cells, which contributes to the activation of HIF-1α. The action of ACF inhibits AKT phosphorylation, possibly due to excessive ROS production (resulting from inhibition of PDK1 transcription) and blockage of the PI3K/PDPK1 pathway. This consequently leads to the death of melanoma cells (Figure 7A).^10^

Other studies also suggest the role of ACF in inhibiting the translation of ATF4, which is the major transcription factor in the UPR (limits cell damage during stress and is induced by hypoxia). This is made possible by blocking elF2α phosphorylation by inhibiting the PERK/eIF2α/ATF4 UPR pathway. As a result, ACF resensitizes the tumor to anticancer therapy.^9^

It has been demonstrated that ACF induces autophagy in the absence of stress related to hypoxia via HIF-1α-dependent as well as HIF-1α-independent pathways, using the MG-63 human osteosarcoma cell lines as an *in vitro* model. It was shown that ACF administered at a dose above 5 μM inhibited cells’ growth and promoted apoptosis of MG-63 cells through the cleavage of PARP-1 (proteins involved in DNA repair) and activation of caspases, responsible for cell destruction during apoprosis, depending on the dose of the drug. It causes the rupture of the mitochondrial membrane, initiating mitochondrial apoptosis. ACF also upregulates Beclin1, Atg5, and LC3-II that have been suggested to inhibit topoisomerases I and II involved in HIF-1α translation.^100^ Similarly, in relation to liver cancer and A549 lung adenocarcinoma,^75^ ACF acts through the caspase-3 activation pathway and cleaves PARP-1.

Despite ample evidence of the effectiveness of ACF, an adequate therapy with this drug is still being sought, e.g., toward pancreatic ductal adenocarcinoma (PDAC). PDAC is characterized by a high degree of hypoxia and a system that protects against drug invasion. Recently a cell culture model was suggested to assess ACF toxicity. It was demonstrated that, in the moderately differentiated PDAC model, ACF inhibited tumor growth; however, unfortunately, it was not observed in the rapidly growing model with high EMT. Moreover, a new metabolic activity of ACF related to the reduction of OXPHOS pathways was detected.^7^

**Figure 8:**
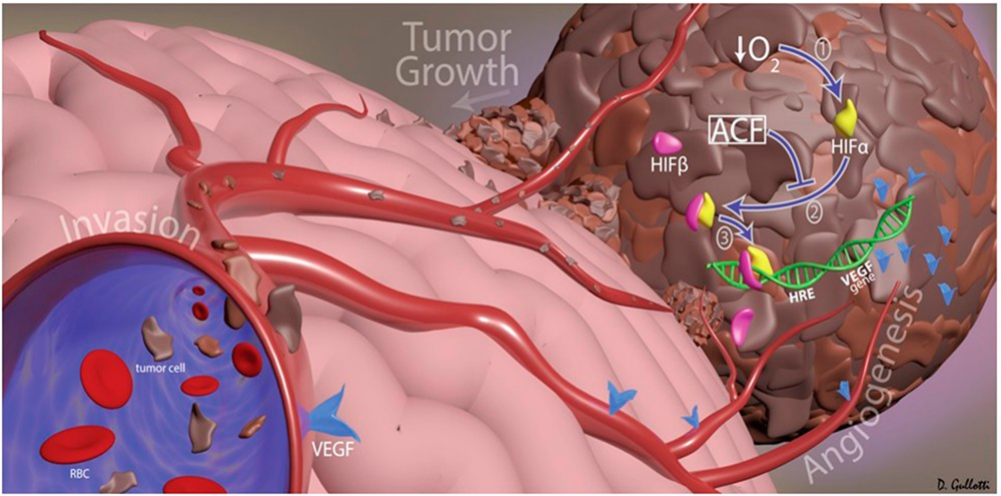
Pathways involved in HIF-1α-mediated glioma tumor formation. Reprinted with permission from ref (39). Copyright 2017 The Authors. Published Open Access by Springer Nature under a Creative Commons CC BY license.

In the case of a brain tumor, there is also a clear association between hypoxia-induced gene overexpression, increased tumor cell invasion, and chemical resistance associated with resistance to apoptosis.^101^ Therefore, also in this case, molecular therapy targeting HIF-1α can be an effective therapeutic option.^102,103^ This is further evidence that HIF-1 plays a significant role in determining the size of brain tumor invasion and, as well, its relapse. Moreover, HIF-1α promotes stabilization of glioblastoma stem cells (GSCs) (Figure 8).^39^ Thus, inhibition of hypoxia is crucial in anti-GSC therapy, especially in the combination therapy with digoxin and ACF.^104^ Considering the poor permeability of the blood–brain barrier for hydrophilic agents, attention was also drawn to the coupling of ACF with the biodegradable polymer poly(1,3-bis[*p*-carboxyphenoxy]propane-*co*-sebacic acid) (p[CPP:SA, 20:80]). In this combination, ACF proved to be effective, being released for over 100 days and achieving almost 100% success in *in vivo* studies in rats with 9L gliosarcoma.^39^

The targeting of tumor stem cells related to the HIF-1 pathway has been noted by Giulia Cheloni et al.^105^ Since CML is caused by hematopoietic stem cells (HSCs) and increased expression of the BCR/Abl tyrosine kinase,^1^ the studies were devoted to a search for effective inhibitors of this kinase and for drugs targeting leukemia stem cells (LSCs), responsible for relapse. *In vitro* tests have shown that greater anti-leukemic efficacy can be achieved by targeting HIF-1α than by blocking the expression of tyrosine kinase. The use of ACF as the main inhibitor of HIF-1 in a mouse CML model resulted in the inhibition of tumor and stem cell growth.^105^

Research on the treatment of leukemia also revealed that HIF-1 is activated not only by hypoxia but also by the regulation of STAT3 and STAT5 (signal transducer and activator of transcription 3 and 5).^106,107^ Since ACF targets both STAT5 and HIF-1 simultaneously, leading to apoptosis of cancer cells, it could create a novel therapeutic approach against leukemia relapse.^71^

Inflammation of the colon caused by hypoxia and the infiltration of macrophages involved in promoting oncogenesis markedly increase the progression of colon cancer (CAC). Relevant studies confirmed the activity of ACF against this type of tumor in immunocompetent mice. Using the tumor allograft model, it was confirmed that ACF treatment inhibited tumor growth through HIF-dependent mechanisms.^38^

ACF was tested against colorectal cancer (CRC) and ovarian cancer (OC) cells, and results were compared with those obtained with standard drugs such as 5-FU, irinotecan, and oxaliplatin in tumor samples from patients.^72^Table 2 shows that ACF was more active against CRC, OC, and chronic lymphocytic leukemia (CLL), compared to other drugs. Unlike ACF, these drugs are also cytotoxic to normal mononuclear cells. It was found that ACF showed also low cross-resistance.

**Table 2:** IC_50_ (μM) Study for Anti-cancer Drugs against Colorectal Cancer (CRC), Ovarian Cancer (OC), and Chronic Lymphocytic Leukemia (CLL) Tumors^72^

drug	CRC	OC	CLL	mononuclear cells
ACF	1.4	4.2	2.6	1.4
5-FU	755.2	562.8	658.2	429.8
irinotecan	89.6	75.3	29.3	25.4
oxaliplatin	26.1	10.9	7.6	2.9

**Figure 9:**
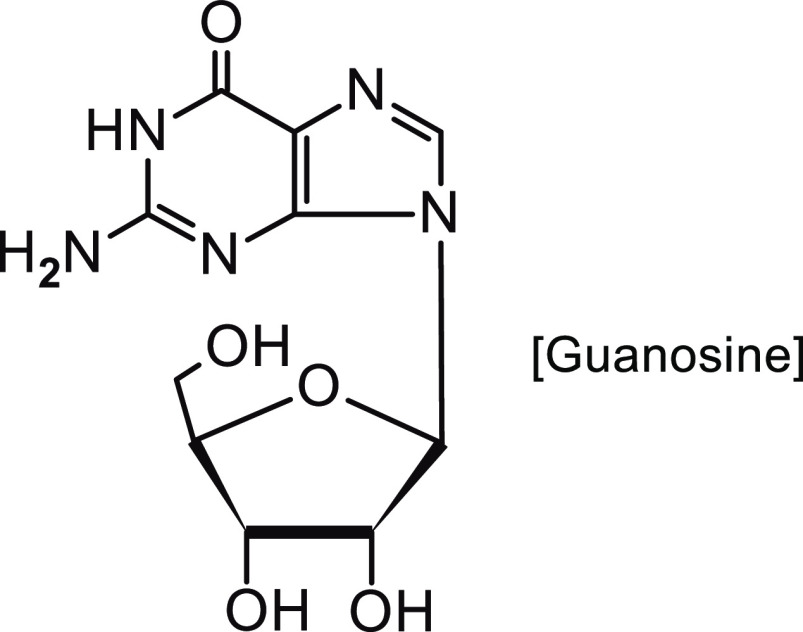
Structure of guanosine.

It turns out that the intravenous (i.v.) route of ACF administration is not the only solution in anticancer therapy. The intramuscular route may be a better method of ACF administration in the form of a mixture with guanosine (molar ratio 1:1) (Figure 9), which enhances the anticancer effect of some drugs.^108^ The suggested effectiveness of the combined use of ACF together with guanosine was presented already in 1996.^109^ The hypothesis that the combined treatment of ACF with guanosine may enhance the antitumor effect was confirmed. ACF interacts with the plasma membrane and modifies the permeability, while guanosine interferes with the production of ATP in the tumor. Research on the ACF-guanosine system was further continued on animal models with subcutaneous Ehrlich carcinoma and intraperitoneal (i.p.) implantation of an Ehrlich ascitic tumor.^109^

ACF may revolutionize the approach not only in the field of radiotherapy or chemotherapy but also in cancer immunotherapy.^110^ The therapeutic benefits of ACF in combination therapy with TRP-2 and anti-PD-1 antibodies have been reported in the treatment of melanoma. The use of this triple-drug system resulted in complete tumor remission, contrary to the data obtained for anti-PD-1 + TRP-2 only.^111^

**Table 3:** Anti-cancer Effect of Acriflavine on Selected Tumors^a^

tumor	cell lines/mice	material/dose	results	ref
**Brain Cancer**	*in vitro*: 9L, GL261, U87, F98, BTSC	10%, 25%, and 50% ACF in ACF:CPP:SA	• local ACF therapy: CPP:SA improves survival	(39)
	*in vivo*: rats with gliosarcoma 9L	*in vivo*: local injections of 5 mg/kg/day	• the optimal dose of ACF is 25% in combination with the polymer CPP:SA	
			• greater efficiency of local ACF delivery compared to systemic administration	

**Pancreatic Cancer (PDAC)**	*in vitro*: Panc-1, THP-1	2.5 μM	• *in vitro*, ACF reduces EMT	(7)
	*in vivo*: mice with PDAC		• *in vitro*, blocks the activity of TGF-β1 associated with the induction of EMT	
			• *in vivo*: ACF did not affect tumor growth in the fast-growing PDTX model (PAC010), but in a relatively slow-growing model (PAC006), ACF showed significant tumor growth reduction and size stabilization	

**Chronic Myeloid Leukemia (CML)**	*in vitro*: K562, KCL22, LAMA-84, HEK293T, and NIH/3T3	*in vivo*: i.p. injections (8 mg ACF/kg/day) for 10 days	• ACF inhibits CML stem cells that are not susceptible to traditional treatment with tyrosine kinase inhibitors	(105)
	*in vivo*: mice C57BL/6J-CD45.1 with CML		• ACF may prevent CML recurrences	
	primary cells of a CML patient			

**Lung Cancer**	*in vitro*: A549	ACF-SLN (ACF DL = 31.25 ± 4.21 mg/mL), 0–14 μM	• ACF-SLN showed a stable cytotoxic effect after 48 h, inducing greater apoptosis compared to the free drug	(85)

**Lung Cancer A549**	*in vitro*: A549	*in vitro*: 0, 1 and 2 μM ACF/48 h	• ACF acts through the caspase-3 activation pathway	(75)
	*in vivo*: nude mice with A549 tumor xenograft (BALB / cAnN.Cg-Foxnl nu/CrlNarl)	*in vivo*: i.p. injection for 6 weeks, 2 mg/kg ACF (60 μL of ACF)	• ACF reduces tumor size *in vivo*	

**Lung Cancer**	*in vitro*: A549	PMONA NPs (microporous silica with cisplatin and ACF)	• ACF increases the anti-tumor efficacy of cisplatin *in vitro*	(96)
	*in vivo*: A549 xenograft mice	*in vitro*: 1-20 μM cisplatin	• PMONA loaded with two drugs had a stronger anti-cancer effect than nanoparticles loaded with one drug	
		PMONA (2 mg cisplatin/kg) DL (% ACF) = 3.2 ± 1.2		

**Hepatocellular Carcinoma (HCC)**	*in vitro*: human HCC cells: Mahlavu, SK-Hep1, Hep3B, Huh-7, and PLC/PRF/5	*in vitro*: 1, 2, 5, and 10 μM	• ACF acts through the caspase-3 activation pathway	(8)
	*in vivo*: Mahlavu cell xenograft mice	*in vivo*: injection of 2 mg/kg daily for 5 weeks	• inhibits the viability of HCC cell lines in a dose-dependent manner	
			• inhibits the growth of neoplastic cells *in vivo*	

**Cervical Cancer**	*in vitro*: HeLa	Nonoplatforma: ACF@PCN-222@MnO_2_-PEG	• enhancement of PDT	(76)
	*in vivo*: female Kunming mouse model with U14 cells			

**Colorectal Cancer (CRC)**	primary tumor cell cultures from patients	*in vitro*	• ACF is more active against CRC (IC_50_ = 1.38 μM) than against OC (IC_50_ = 4.23 μM) and CLL (IC_50_ = 2.58 μM)	(72)
			• ACF is an inhibitor of topoisomerases I and II	

**Colitis-Associated Colon Cancer (CAC)**	mice Balb/C	*in vivo*: injections 2 mg/kg/day	• ACF reduces vascularity growth and tumor progression	(38)
			• ACF acts on HIF-1	

**Colorectal Cancer (CRC)**	SW480, HCT116, LS174T	*in vitro*: 0.07, 0.15, 0.31, 0.62, 1.25, 2.5, and 5 μM/72 h	• ACF enhances the effect of 5-fluorouracil better than irinotecan	(79)
			• it exhibits a different mechanism than the suppression of HIF-1α and topoisomerase II expression (their levels were unchanged)	

**Colorectal Cancer**	*in vitro*: CT26	DOX-ACF@Lipo (encapsulated DOX and ACF in liposomes)	• DOX-ACF@Lipo cellular uptake is dependent	(8)
	*in vivo*: Balb/c mice with the CT26 tumor	*in vitro*: DOX-ACF@Lipo and DOX@Lipo ([DOX] = 0.047, 0.236, 0.47, 0.94, 2.36, and 4.7 μg/mL, [ACF] = 0.1, 0.5, 1, 2, 5, and 10 μg/mL)/24 h	• a better therapeutic effect was achieved by DOX-ACF@Lipo at different concentrations compared to DOX@Lipo	
		*in vivo*: i.v. injections of 5 mg/kg	• *in vivo*: DOX-ACF@Lipo, tumor volume was 28.9%; DOX@Lipo, tumor volume was 32.6%	

**Colorectal Cancer**	*in vitro*: CT26	ACF@MnO_2_	• ACF@MnO_2_ can reduce cell viability more effectively than free acriflavin or free MnO_2_ in the presence of X-rays, significantly less metastasis in the liver was observed	(92)
	
**Breast Cancer**	*in vivo*: mice with 4T1	i.v. injection, 3 mg/kg/14 days	• ACF@MnO_2_ can effectively suppress the expression of metastatic proteins (VEGF and MMP-9)	

**Breast Cancer**	MDA-MB-231, MDA-MB-435	*in vivo*: 4 mg/kg/day i.p.	• ACF acts on HIF-1 by reducing the expression of LOX and LOXL proteins (responsible for metastasis), destroying metastatic niches of breast cancer	(80)
	mice with MDA-435			

**Breast Cancer**	mouse breast cancer cells (4T1 cells)	CSP-ACF nanoparticles	• very low drug concentration (5 μg /mL) in the form of CSP nanoparticles can lead to apoprosis of more than 60% of cancer cells	(74)
		*in vitro*: 0–5 μg/mL	• ACF alleviates hypoxia and makes a patient more sensitive to radiotherapy	
			• CSP-ACF nanoparticles lead to a decrease in VEGF, fewer tumor microvessels and more cell apoptosis	

**Breast Cancer**	*in vitro*: 4T1	ACF-LNC	• higher efficiency of ACF-LNC compared to free ACF	(83)
	*in vivo*: mice with 4T1	*in vivo*: 5 mg/kg	• the use of ACF-LNC allowed reduction of the number of administrations compared to free ACF (from 12 to 2 injections) *in vivo*	

**Breast Cancer**	mice BALB/c with 4T1	*in vivo*: ACF 2 mg/kg i.p.	• ACF increases the antitumor activity of sunitinib, lowers the expression of VEGF and TGF-β, and reduces tumor vascularization, leading to its apoptosis	(78)

**Melanoma**	B16-F10 and 4T1	5, 10, 20, and 30 μM	• ACF improved the effectiveness of cancer immunotherapy in combination therapy with TRP-2 and anti-PD-1 antibody	(111)

**Melanoma**	SK-MEL-28, IGR37, and B16/F10 murine melanoma cells	*in vitro*: 0, 2.5, and 5 μM	• ACF induces melanoma cell death under conditions of normoxia	(10)
			• ACF disrupts glucose metabolism by down-regulating PDK1	
			• inhibits the phosphorylation of AKT and RSK2	
			• targets the activation of transcription factor 4 (ATF4)	
			• inhibits the expression of the transcription factor MITF (the factor responsible for the acts of induction of HIF-1 transcription)	

**Perihilar Cholangiocarcinoma**	SK-ChA-1		• liposomal ACF sensitizes tumor cells to PDT	(73)
			• ACF inhibits HIF-1 and topoisomerases I and II	

**Epidermal Cancer**	A431	*in vitro*: ACF encapsulated in the aqueous core of the liposomes containing the ZnPC photosensitizer	• action of free or liposomal ACF improves the efficacy of PDT	(86)

**Osteosarcoma**	MG63	*in vitro*: 0, 0.1, 1, 5, and 10 μM	• ACF (0–10 μM) inhibits the growth of osteosarcoma cells in a dose-dependent manner	(100)
			• ACF induces tumor apoptosis via both HIF-1α-dependent and HIF-1α-independent pathways	

a Abbreviations used: F98, 9L, GL261, and U87, human glioma cell lines; BTSCs, human primary brain tumor stem cells; CPP:SA, biodegradable polyanhydride poly(1,3-bis[*p*-carboxyphenoxy]propane-*co*-sebacic acid); Panc-1, human pancreatic cancer cells; THP-1, human monocytic cell line; EMT, epithelial-to-mesenchymal transition; PDTX, human PDAC xenografts: PAC006 (classical type, moderately differentiated and slow progression) and PAC010 (quasi-mesenchymal type, poorly differentiated and faster growth); K562, human erythroleukemic cell line; KCL22, human myeloid leukemia cell line; LAMA-84, human chronic myeloid leukemia cell line; HEK293T, human embryonic kidney 293 cells; NIH/3T3, cell lines of mouse embryonic fibroblasts; CML, myeloid leukemia; A549, adenocarcinomic human alveolar basal epithelial cells; ACF-SLN, solid lipid nanoparticles containing ACF; PMONA, cisplatin microporous organosilica nanoparticles with ACF; Mahlavu, SK-Hep1, Hep3B, Huh-7, and PLC/PRF/5, human hepatocellular carcinoma cells; HeLa, epitheloid cervical carcinoma; SW480, human colon adenocarcinoma; HCT116, human colon cancer cell line; LS174T, human intestinal cell line; DOX, doxorubicin; CT26, murine colorectal carcinoma cell line; 4T1, breast cancer cell line; VEGF, vascular endotherial growth factor; MMP-9, matrix metalloproteinase 9; MDA-MB-231 and MDA-MB-435-human breast adenocarcinoma; LOX, lysyl oxidase proteins; LOXL, lysyl oxidase-like proteins; CSP, Cu_2-*x*_Se@PtSe, type of yolk–shell nanosensitizer; ACF-LNC, lipid nanocapsules containing acriflavine; TGF-β, transforming growth factor beta; B16-F10, mouse melanoma cells; TRP-2, tyrosinase-related protein-2; PD-1, programmed death receptor 1; SK-MEL-28 and IGR37, human melanoma cells; PDK1, pyruvate dehydrogenase kinase 1; AKT, protein kinase; RSK2, serine/threonine kinase ribosomal S6 kinase 2; ATF4, activating transcription factor 4; MITF, microphthalmia-associated transcription factor; SK-ChA-1, human cholangiocarcinoma cells; A431, squamous carcinom cell line; MG63, human osteosarcoma cell line; i.p., intraperitoneal; i.v., intravenous.

## 4 Acriflavine as a Drug Inhibiting SARS-CoV-2

The coronavirus COVID-19 pandemic broke out in 2019, when the infectious SARS-CoV-2 virus caused acute respiratory distress syndrome (ARDS) and many other side effects, often leading to death. The identified coronavirus (SARS-CoV-2) is much more contagious compared to other previously identified coronaviruses: SARS-CoV and MERS-CoV. To date, several vaccines and antiviral drugs targeting the coronavirus RNA polymerase (e.g., Remdesivir) have been suggested. Despite the implemented therapies, there is still an intensive search for an effective drug for COVID-19 therapy that will be effective against the mutating SARS-CoV-2 virus.^112^

**Figure 10:**
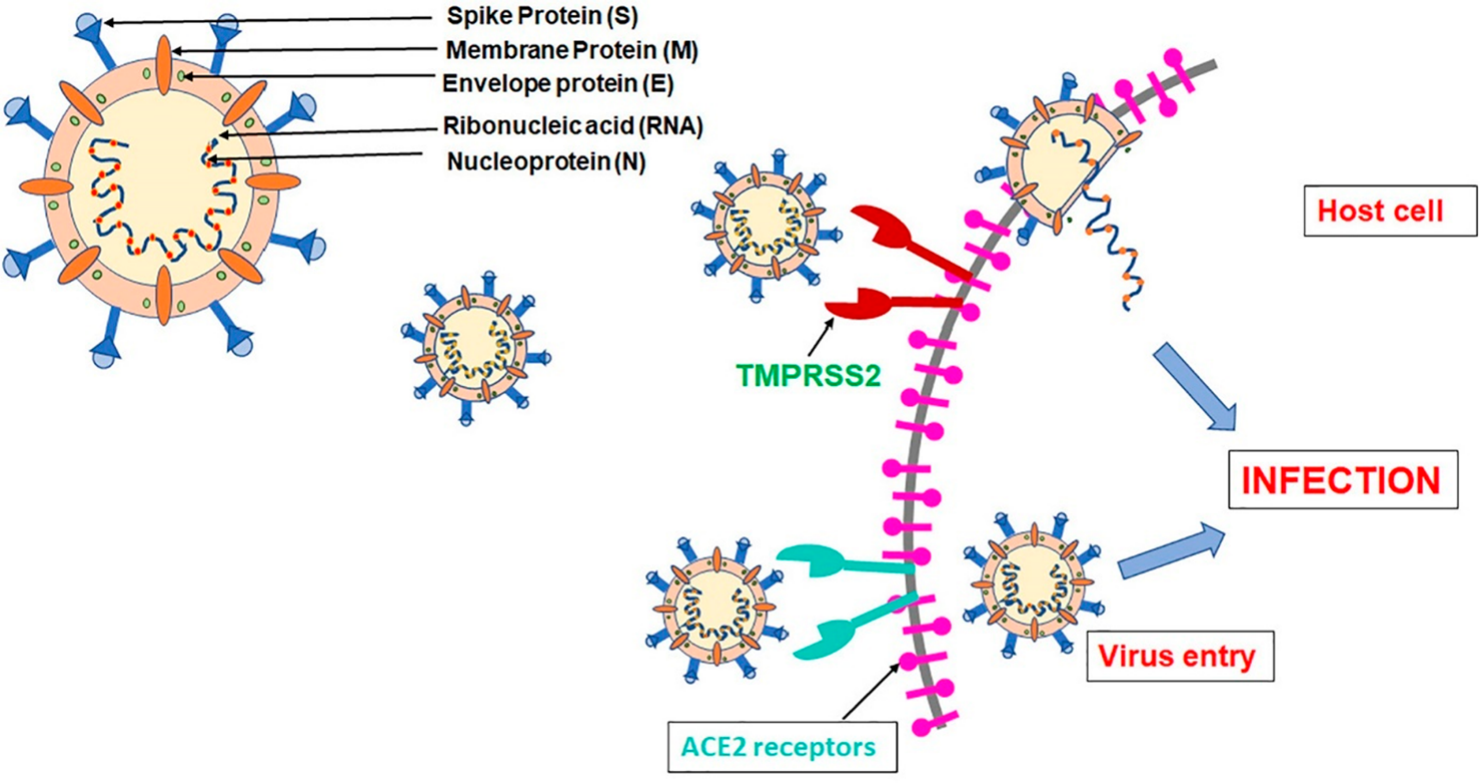
Graphical illustration of the SARS-CoV-2 attack on host cells. Reprinted with permission from ref (113). Copyright 2022 Elsevier.

The lungs, heart, kidneys, intestines, and other organs have ACE2 enzymes on the surface of the cell membrane that act as a receptors, facilitating the entry of SARS-CoV-2 (SARS-CoV-2 S protein interacts with ACE2). Virus multiplication is mediated by M^pro^ and PL^pro^ proteases—coronavirus enzymes. In addition to ACE2, there is also TMPRSS2, a serine protease that facilitates binding of ACE2 to the viral protein. Targeting the M^pro^ and PL^pro^ enzymes could be a major therapeutic pathway for combating coronavirus disease (Figure 10).^113−115^ M^pro^ inhibitors have already undergone clinical trials (NCT04535167, NCT04627532), while the inhibitor of PL^pro^, ACF, has been proposed recently as an effective anti-COVID drug.^18^

Out of 11 compounds chosen for the studies, ACF turned out to be the most active against PL^pro^ (IC_50_ = 1.66 μM). However, such activity was not observed for M^pro^. ACF specifically inhibits the active site of the PL^pro^ enzyme by blocking viral infection in the assay of cell lines: A549 with ACE2 overexpression (CC_50_ = 3.1 μM, IC_50_ = 86 nM), Vero (CC_50_ = 3.4 μM, IC_50_ = 64 nM), HCT-8 (CC_50_ = 2.1 μM), and HSF (primary human fibroblasts, CC_50_ = 12 μM).^18^

It was also noticed that, compared to the currently used remdesivir, ACF is more effective in inhibiting SARS-CoV-2. But in combination therapy of the two drugs, a study on Vero lines showed increased efficacy against SAR-CoV-2 compared to free drugs. Similarly, the superiority of the use of ACF was confirmed in an *ex vivo* human epithelial culture model (HAE) study, while the use of remdesivir requires much higher doses. Moreover, ACF can be administered orally, achieving good therapeutic effectiveness in the lungs.^18^

**Figure 11:**
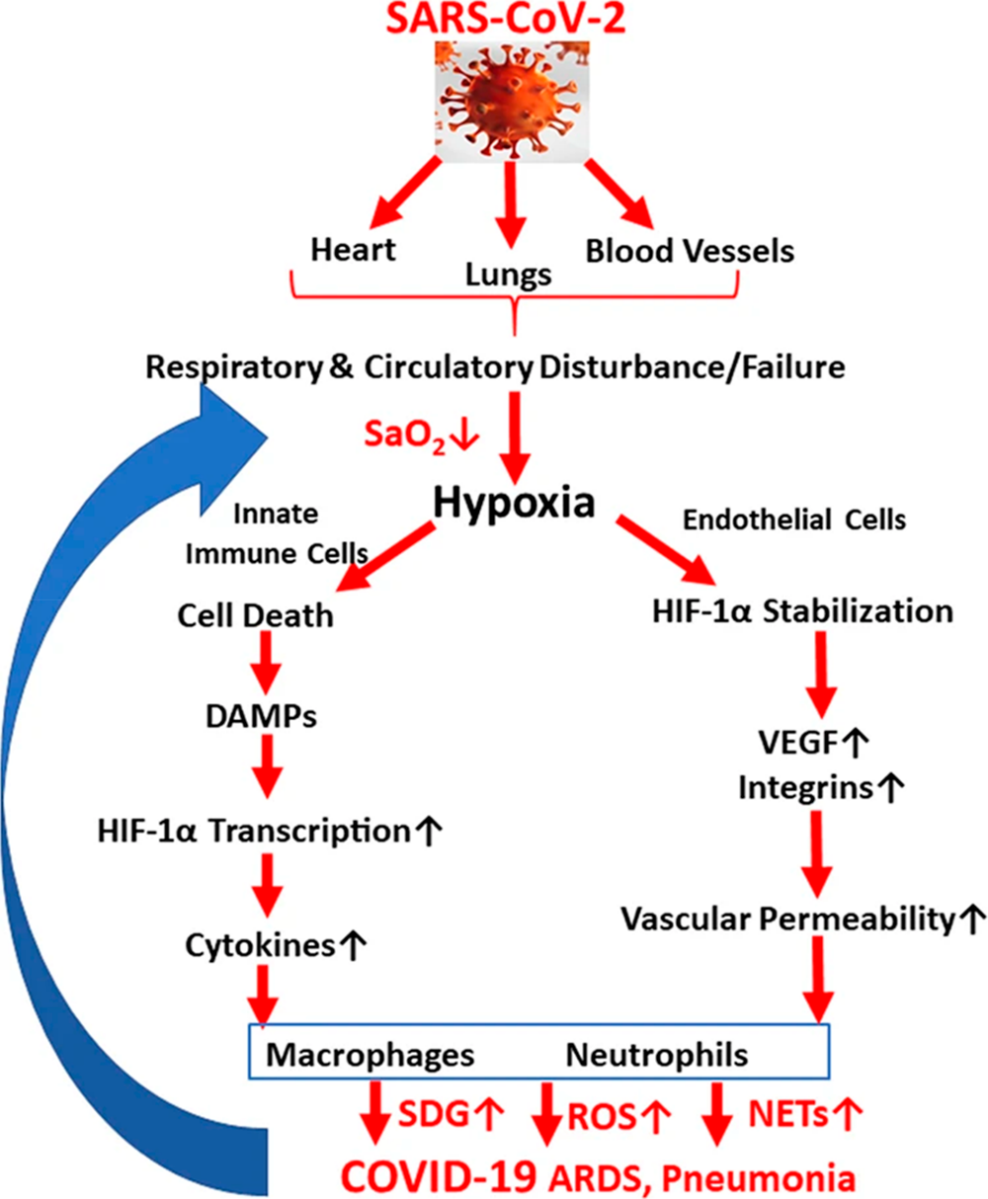
Diagram of SARS-CoV-2 virus entry into the cell after activation of hypoxia-related pathway cells. Reprinted with permission from ref (116). Copyright 2022 Springer Nature.

The proposed mechanism of action of ACF suggests that this drug inhibits the virus at all stages of infection because it affects the replication process and not only the entry of the virus into the host cell.^18^ However, with reference to other literature data, it appears that the effect of ACF on SARS-CoV-2 can be explained not only by targeting the PL^pro^ enzyme but also by the effect on the hypoxia-induced factor HIF-1α (see section 1). This factor can stimulate a “cytokine storm” that leads to organ failure.^116^ When cells become infected with SARS-CoV-2, there is increased expression of HIF-1α, which targets ACE2 and controls viral entry into cells.^117^ There are also suggestions that the stabilization or even activation of HIF-1α, leading to a reduction in SARS-CoV-2 invasiveness, is associated with lowering ACE2 levels as a result of hypoxia (Figure 11).^116,118^ It was ultimately proven that this factor is responsible for the inflammatory process that occurs after infection with the virus.^119^ On the other hand, ACF has been proven to exert HIF-1α-inactivating effect, which may account for the satisfactory results and positively affect COVID-19 therapy.^18^

In addition, it has been proven that PL^pro^ suppresses interferon type I responses, while ACF has properties that engage interferon in the induction of antiviral genes (see section 5),^3^ which may be another pathway accounting for the effectiveness of this drug against COVID-19 and its multitasking nature.

## 5 Other Uses of Acriflavine

Malaria is an infectious disease caused by *Plasmodium* parasites that attacks red blood cells. The problem in the effective treatment of this disease is resistance to the drugs used so far.^120^ The antimalarial activity of ACF was first demonstrated in 2014 by scientists from India, who based their conclusion on previous studies reporting the antimalarial properties of acridine derivatives.^12,121^ ACF was found to kill *Plasmodium falciparum* malaria parasites *in vitro*, including those resistant to chloroquine, and to act *in vivo* in a mouse model system against the rodent-specific parasite *Plasmodium berghei*. Additionally, ACF was active at all three stages of the *P. falciparum* life cycle. It has also been shown to be specifically accumulated in infected red blood cells and not in the uninfected ones, possibly due to the presence of some parasite-specific transporters that capture ACF.^12^ The effectiveness of ACF in combating other parasitic diseases, e.g., *Centrocestus formosanus* and *Trichodia centrosrigeata* affecting the gills of *Oreochromis niloticus* fish,^122^ has also been confirmed.

ACF also works against *Acanthamoeba*, which is a protozoan that causes an infection of the cornea of the eye and even granulomatous encephalitis.^123^*Acanthamoeba* causes infection, and it remains in the form of a trophozoite and undergoes mitosis. It shows high resistance to drugs, with the ability to transform into a dormant form of cysts.^124^ Studies were also devoted to the action of ACF against three strains of *Acanthamoeba* of different pathogenicity and showed that ACF works even on resistant protozoan cysts and destroys trophozoite within 24 h.^123^

ACF intercalates DNA, which may contribute to the fight against *Trypanosoma cruzi*. It leads to changes in the kDNA structure of this protozoan, which results in the formation of dyskinetoplastic (Dk) strains and, consequently, inhibition of infection.^125^

There was also suggested a different mechanism of action of ACF on pathogens. Based on conclusions from several previous studies,^126−128^ it was shown that *in vivo* injection of ACF induced interferon-like activity in the serum of mice. Further studies have shown that other acridines engage type I interferon signaling and protect infected cells from infection, thanks to the interferon gene stimulator (STING) that is involved in detection of cytosolic DNA and promotes the induction of antiviral genes. It has been shown that the mixture of acriflavine and proflavine, which causes low levels of DNA damage and cytoplasmic DNA leakage, activates cGAS-dependent STING and thus has antiviral properties in human cells.^3^ ACF clinical trials demonstrating its antiviral properties were already known in the 1990s in the context of anti-HIV activities, when used in commbination with other drugs.^14,70,129,130^

The action of ACF may be also significant in combating the fungal infections from *Trichophyton rubrum* (fungus affecting keratinized tissues)^16^ and *Candida utilis* yeasts.^131,132^ Recent studies suggest that ACF induces changes in the structure of the catalase enzyme, which in turn cause apoptosis and yeast necrosis.^133^

ACF also exhibits antibacterial properties. ACF was reported to be effective against *Rhinoscleroma* in the 1980s,^134^ and in the late 1990s, its antiseptic properties for mouthwash were demonstrated.^135^ The renewed interest in this acridine derivative is associated with an increase in drug-resistant bacterial infections. Research results show that ACF hydrochloride may be effective in treatment of *Helicobacter pylori* infection. This pathogen increases the risk of stomach cancer and is resistant to most antibiotics used.^136^ ACF·HCl binds to the proteins of the pathogen’s cell membrane and inhibits its growth.^137^ This translates into sensational research results that suggest the complete elimination of *H. pylori* from the stomach tissues of ACF·HCl-treated mice in *in vivo*. A strong synergistic effect of ACF hydrochloride with clarithromycin on inhibiting the growth of these bacteria has also been demonstrated.^137^

**Figure 12:**
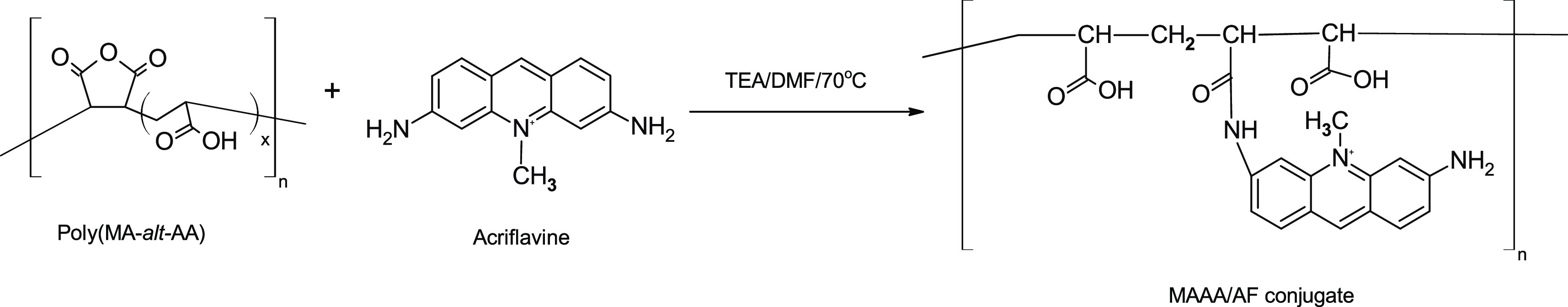
Scheme of synthesis of acriflavine conjugate with poly(maleic anhydride-*alt*-acrylic acid) copolymer (MAAA).

Other studies describe the effectiveness of the ACF delivery system using the carrier poly(maleic anhydride-*alt*-acrylic acid) copolymer (MAAA). ACF was covalently bound to the carrier. In this combination, it showed a stronger antibacterial activity against enterohemorrhagic *Escherichia coli* (EHEC) and *Staphylococcus aureus* compared to free ACF (Figure 12).^138^

Today, ACF is still used in Asia as a topical antiseptic against Gram-positive and Gram-negative bacteria.^20^

Unfortunately, it turns out that some bacteria have developed a defense mechanism against ACF.^139^ An example is *Staphylococcus aureus*, from which a particular mutant is derived, 209P, which shows a significant thickening of the cell walls after ACF treatment.^140^ There are also reports concerning drug-developed resistance to other bacteria, such as *E. coli* K-12.^141^ Due to reports describing the development of resistance by some bacteria to ACF, ACF is more and more often referred to in the context of its strong anticancer and antiviral properties and as a potential drug against SARS-CoV-2.

## Conclusion

In the present work, the latest research directions involving acriflavine (ACF), its complexes and conjugates, and their mechanism of action have been presented. For years such systems were and still are known as antibacterial drugs and prodrugs. However, currently most of the research efforts concentrate on prodrug systems to be applied in anticancer therapy as well as potential antiviral agents, e.g., inhibiting SARS-CoV-2.

As far as anticancer properties of ACF are regarded, one should notice its tumor-reducing action via caspase-3 activation in lung cancer cases, but the whole spectrum of its superior activity was proven against colorectal, ovarian, breast, and cervical cancer cells. ACF effectively intercalates with DNA. As a result, it has the ability to interfere with many cellular functions. ACF acts as an inhibitor of protein kinases, topoisomerases I and II, and hypoxia-induced factor 1α (HIF-1α) and also leads to upregulation of genes, especially long non-coding RNAs (lncRNAs). ACF was also found to be effective in combination therapy, e.g., with doxorubicin, cisplatin, and 5-fluorouracil, as well as in radiotherapy and PDT. The free ACF is a potent but short-lived species due to its fast metabolism, and as a small molecule, it is relatively quickly cleared from the body. A number of nanoplatforms have been studied, including liposomes, polymers, and nanosilica, allowing its *in vitro* for release up to 60 days.

It was recently found that, compared to the currently used remdesivir, ACF is more effective in inhibiting SARS-CoV-2, and in combination therapy with the two drugs, a study on Vero lines showed increased efficacy against SAR-CoV-2 compared to free drugs. The efficacy of ACF was also confirmed in an *ex vivo* human epithelial culture model (HAE) study, while the use of remdesivir requires much higher doses. Moreover, ACF is active as an antimalarial, antibacterial, antiviral (HIV), antituberculosis, and fungicidal. Thus, although we are dealing with an old drug structure, it appears as a newly revisited field of application against most serious contemporary diseases.

## Abbreviations Used

5-FU: fluorouracil
4T1: breast cancer cell line
A431: squamous carcinom cell line
A549: adenocarcinomic human alveolar basal epithelial cells
ACE2: angiotensin-converting enzyme 2
ACF: acriflavine
ACF-LNC: lipid nanocapsules containing acriflavine
ACF-SLN: solid lipid nanoparticles containing acriflavine
aFGF, bFGF: acidic and basic fibroblast growth factors
AKT: protein kinase
AML: acute myeloid leukemia
Ang2: angiopoietin-2
AT1: angiotensin-1
ATF4: activating transcription factor 4
AT2: angiotensin-2
ATP: adenosine triphosphate
Atg5: autophagy related 5
B16-F10: mouse melanoma cells
BCL-B: cell lymphoma
BTSCs: human primary brain tumor stem cells
CAV-1 and CAV-2: caveolin-1 and -2
CAF: cancer-associated fibroblasts
CAC: colon cancer
CLL: chronic lymphocytic leukemia
CPP:SA: biodegradable polyanhydride poly(1,3 bis[*p*-carboxyphenoxy]propane-*co*-sebacic acid)
CML: myeloid leukemia
CT26: murine colorectal carcinoma cell line
CSP: Cu_2–*x*_Se@PtSe, a type of yolk–shell nanosensitizer
CSCs: cancer stem cells
DNA: deoxyribonucleic acid
DOX: doxorubicin
Dk: dyskinetoplastic
eIF2α: eukaryotic initiation factor 2α
ERK: extracellular-regulated kinase
EMT: epithelial-to-mesenchymal transition
EGF: epidermal growth factor
EGFR: epidermal growth factor receptor
FDA: U.S. Food and Drug Administration
F98, 9L, GL261, and U87: human glioma cell lines
GM-CSF: granulocyte-macrophage colony-stimulating factor
GLUT-1: glucose transporter 1
GSCs: glioblastoma stem cells
HIF-1α: hypoxia-induced factor 1α
HGF: hepatocyte growth factor
HCC: hepatocellular carcinoma
HSCs: hematopoietic stem cells
HAU: human epithelial culture model
HCT116: human colon cancer cell line
HEK293T: human embryonic kidney 293 cells
HeLa: epitheloid cervical carcinoma
lncRNAs: long non-coding ribonucleic acids
IL-1: interleukin-1
IL-6: interleukin-6
IL-8: interleukin-8
IL-10: interleukin-10
IL-12: interleukin-12
INF-α: interferon-α
K562: human erythroleukemic cell line
KCL22: human myeloid leukemia cell line
LNCs: lipid nanocapsules
LSCs: leukemia stem cells
LAMA-84: human chronic myeloid leukemia cell line
LS174T: human intestinal cell line
LOX: lysyl oxidase proteins
LOXL: lysyl oxidase-like proteins
MET: mesenchymal-to-epithelial transition
MITF: microphthalmia-associated transcription factor
MCL-1: myeloid leukemia 1
M^pro^ and PL^pro^: main protease and papain-like protease
MAAA: poly(maleic anhydride-*alt*-acrylic acid) copolymer
Mahlavu, SK-Hep1, Hep3B, Huh-7, and PLC/PRF/5: human hepatocellular carcinoma cells
MMP-9: matrix metalloproteinase 9
MDA-MB-435: human breast adenocarcinoma
mTOR: mammalian target of rapamicin
MG63: human osteosarcoma cell line
NO: nitric oxide
NV: neovascularization
NIH/3T3: cell lines of mouse embryonic fibroblasts
Oct-3/4: octamer-binding transcription factor 3/4
OXPHOS: mitochondrial oxidative phosphorylation system
OC: ovarian cancer
PERK: protein kinase RNA-like endoplasmic reticulum kinase
PDGF: plated-delivered endothelial growth factor
PGE: prostaglandin E
PAI-1: prasminogen activator inhibitor-1
PI3K: phosphoinositide 3-kinases
PD-L1: programmed cell death ligand 1
PLGA: poly(lactic-*co*-glycolic acid)
PTX: paclitaxel
PDT: photodynamic therapy
PARP1: poly[ADP-ribose]polymerase 1
PDAC: pancreatic ductal adenocarcinoma
Panc-1: human pancreatic cancer cells
PDTX: human PDAC xenografts: PAC006 (classical type, moderately differentiated, and slow progression) and PAC010 (quasi-mesenchymal type, poorly differentiated, and faster growth)
PMONA: cisplatin microporous organosilica nanoparticles with ACF
PD-1: programmed cell death protein 1
PDK1: pyruvate dehydrogenase kinase 1
RNA: ribonucleic acid
ROS: reactive oxygen species
RSK2: serine/threonine kinase ribosomal S6 kinase 2
Sox-2: sex-determining region Y-box 2
SARS-CoV-2: severe acute respiratory syndrome coronavirus 2
SW480: human colon adenocarcinoma
SK-MEL-28 and IGR37: human melanoma cells
SK-ChA-1: human cholangiocarcinoma cells
STAT3 and STAT5: signal transducer and activator of transcription 3 and 5
TNF-α: tumor necrosis factor-α
TIMPs: tissue inhibitor metalloprotease
TNBC: triple-negative breast cancer
THP-1: human monocytic cell line
TGF-β: transforming growth factor beta
TRP-2: tyrosinase-related protein-2
UPR: unfolded protein response
VEGF: vascular endothelial growth factor
